# Left Atrial Masses: Now You See Them, Now You Don't

**DOI:** 10.7759/cureus.50056

**Published:** 2023-12-06

**Authors:** Gaayathri Krishnan, Denham Windross, Shahnaz Punjani, Chad Brands, Thomas Shimshak

**Affiliations:** 1 Medicine, AdventHealth Sebring, Sebring, USA; 2 Internal Medicine, PSG Institute of Medical Sciences and Research (IMSR), Coimbatore, IND; 3 Internal Medicine, AdventHealth Sebring, Sebring, USA; 4 Interventional Cardiology, AdventHealth Florida, Sebring, USA

**Keywords:** atrial fibrillation, thrombus, cardiac, myxoma, left atrial mass

## Abstract

Left atrial masses are rare but clinically significant findings, which can present as diverse pathological entities, including primary tumors, thrombi, and metastases. Their diverse pathological entities contribute to a wide range of clinical manifestations, often presenting with nonspecific symptoms that pose challenges for early diagnosis.

Within the realm of medicine, unique presentations emphasize the intricate interplay between the size, location, and functional impact of pathological processes. They serve as reminders to healthcare providers to approach each patient as an individual, recognizing that even seemingly minor abnormalities can have significant consequences.

To illustrate this, we present two distinct cases of patients with left atrial masses, showcasing the importance of clinical suspicion and a wide knowledge base in identifying and managing these conditions effectively.

## Introduction

Left atrial masses encompass a diverse range of pathologies, including both neoplastic and non-neoplastic entities. Primary cardiac tumors are rare, with a reported frequency of 0.02% based on postmortem studies. Approximately 75% of primary tumors are benign, and 50% of benign tumors are myxomas, resulting in 75 cases of myxoma per million autopsies [1,2].

To accurately diagnose the origin of left atrial masses, various imaging techniques are employed, including echocardiography, computed tomography (CT), and magnetic resonance imaging (MRI). The specific characteristics of the mass, such as its location, shape, and vascularity, along with clinical presentation and patient history, help guide the diagnosis and subsequent management, which may involve surgical removal, medical treatment, or interventions to prevent embolization or complications.

We present two patients with incidentally identified left atrial masses and summarize their vastly different clinical courses.

## Case presentation

Case 1

**Figure 1:**
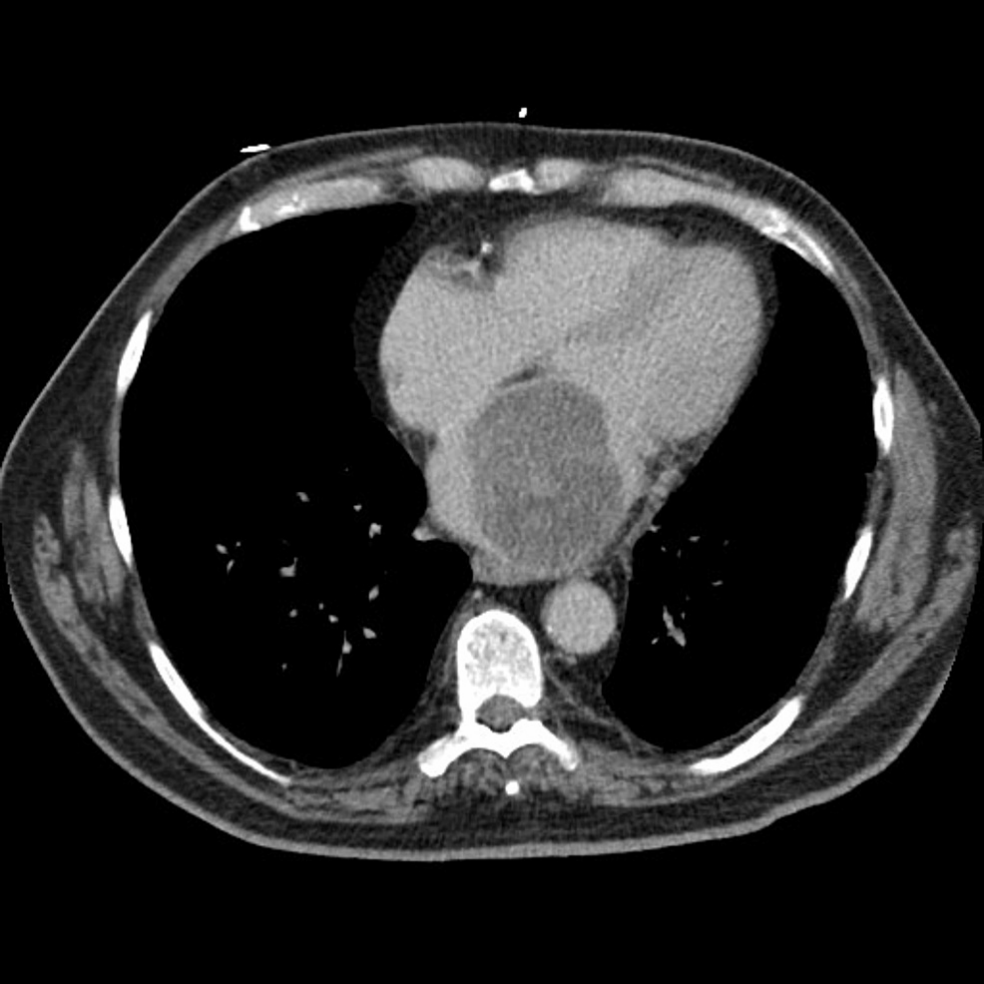
Left atrial filling defect noted on CT abdomen pelvis

A 68-year-old asymptomatic man underwent routine CT of the abdomen and pelvis (Figure 1) prior to undergoing elective herniorrhaphy. A large filling defect of the left atrium was identified. A physical examination including the cardiovascular system proved benign, exhibiting no murmurs or added heart sounds.

**Figure 2:**
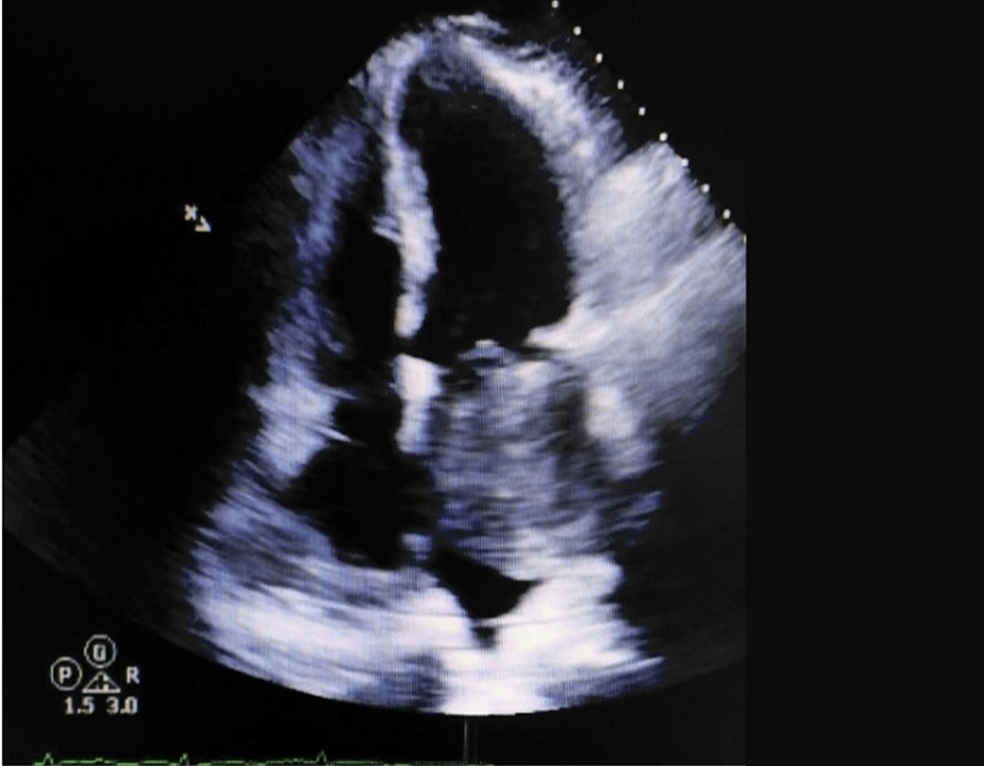
Transthoracic echo with left atrial mass

Subsequent two-dimensional transthoracic echocardiogram revealed a 6 cm x 7.3 cm mass in the left atrium, consistent with a left atrial myxoma (Figure 2). Although he was asymptomatic, surgical intervention was recommended. He underwent elective preoperative cardiac catheterization and coronary angiography, demonstrating complex multivessel disease. He ultimately underwent elective bypass surgery and excision of the left atrial mass. Histopathologic examination of the mass confirmed the diagnosis of left atrial myxoma.

Case 2

**Figure 3:**
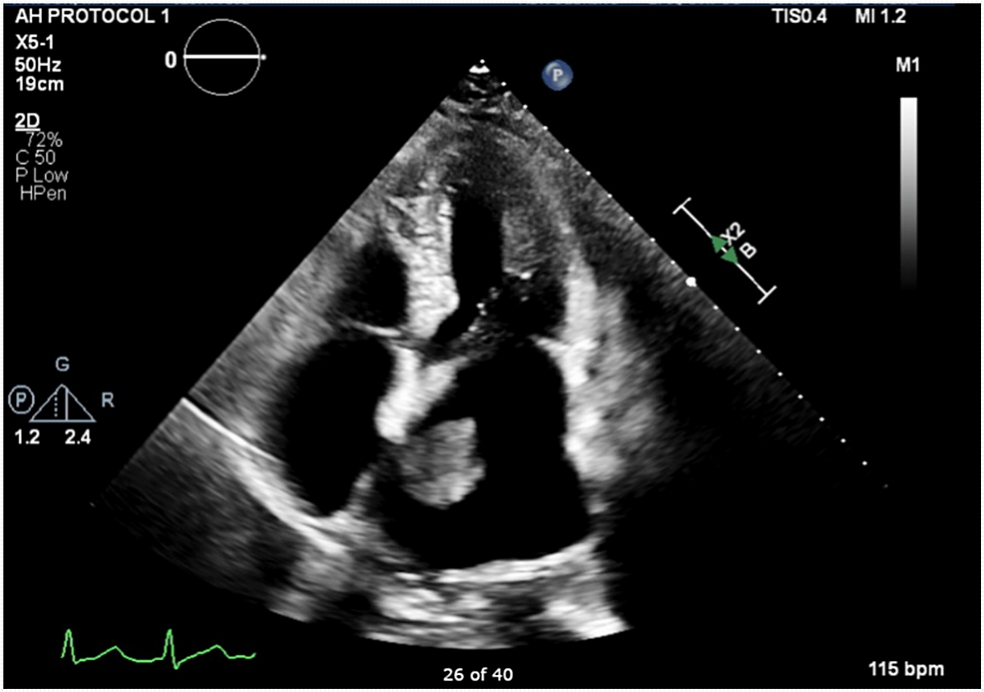
Transthoracic echo with left atrial mass

An 83-year-old female presenting to the emergency room with fatigue was admitted to the hospital for micro-atrioventricular (AV) pacemaker implantation and AV nodal ablation to treat persistent atrial fibrillation. An elective transthoracic echocardiogram (Figure 3) revealed a dilated left atrium with a 2.48 cm x 2.49 cm mobile mass attached to the atrial septum with a stalk.

After much deliberation, a conclusion arrived that the mass was most probably a left atrial myxoma owing to the size, location, and appearance, and thus, she underwent the procedure and was planning to be discharged home since she was clinically stable and tolerated the procedure well.

**Figure 4:**
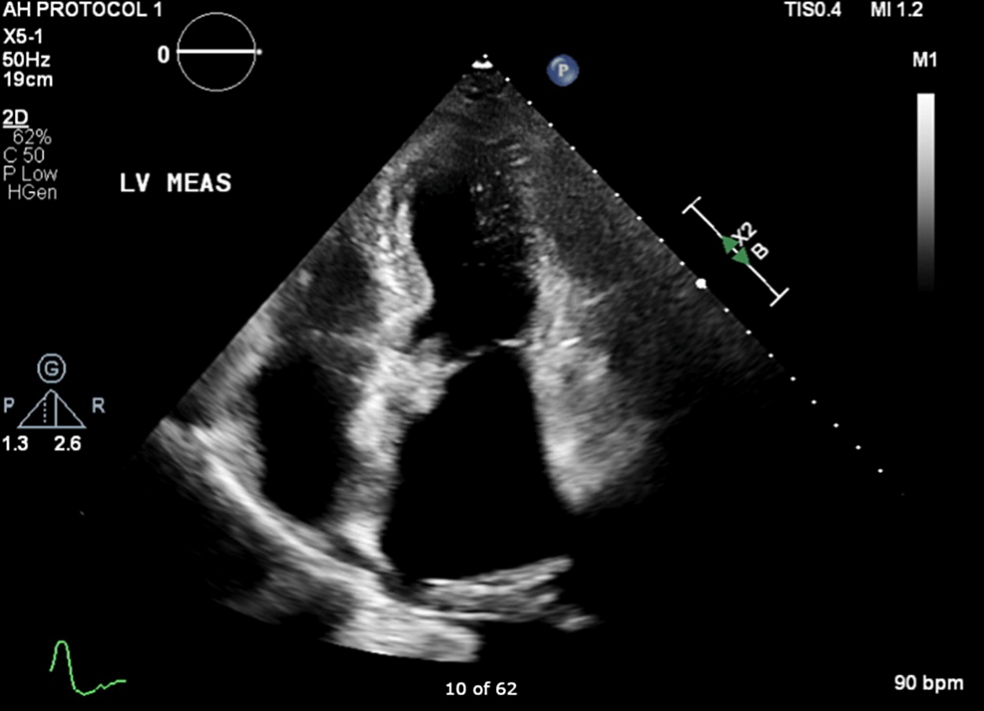
Repeat transthoracic echo without the left atrial mass

A rapid response was called suddenly because the patient became acutely hypotensive. Although the patient remained at her baseline mentation, a manual blood pressure reading on her left arm was unobtainable. In addition, her arm was cool to touch and pale, with absent pulses. Her other hand, however, exhibited a normal examination with full pulses and normal blood pressure. An urgent repeat echocardiogram revealed a complete absence of the previously identified left atrial mass (Figure 4). Physical findings were consistent with acute ischemia of the left arm.

An emergent, selective arteriogram of the left upper extremity was then performed via the right femoral artery, demonstrating an extensive, obstructive thrombus beginning at the level of the brachial artery and extending distally to involve her hand. 

The extent of the thrombus and her extensive ischemia of the left arm precluded endovascular therapy. She underwent a successful emergent embolectomy/thrombectomy with complete restoration of her left arm and hand perfusion and resolution of her symptoms.

## Discussion

Myxomas can lead to severe complications but have an excellent prognosis, with operative mortality not exceeding 5% when detected and treated early [3].

Most patients with atrial myxoma present with one or more of the characteristic triad of symptoms: symptoms of obstruction leading to left or right heart failure signs, peripheral thromboembolisms, or constitutional symptoms [4-6]. Symptomatic status is dependent on the size, location, and mobility of the tumor. In one-third of patients with myxoma, physical exams can reveal an early diastolic murmur, the classic “tumor plop” [7].

Unique presentations in medicine often highlight the intriguing nature of the human body and its complex mechanisms. It is fascinating how something seemingly insignificant or small can manifest as a significant clinical finding, while something large can remain asymptomatic. Tumors as large as those in this first case are rarely asymptomatic [2,3,8]. Thus, this warrants awareness and knowledge of such tumors.

The second patient’s clinical course was vastly different, with significant embolization resulting in acute ischemia of the left upper extremity. In medicine, the concept of "small but significant" is exemplified by certain conditions or abnormalities such as this patient's clinical scenario.

These deeply contrasting cases are eye-opening to the wonder that is the human body and the fact that a small chamber, less than 4 cm (about half the length of the long edge of a credit card) could harbor such different pathologies.

Understanding the variability in clinical presentations and the unpredictable nature of disease progression is vital in providing comprehensive medical care. By remaining vigilant to both subtle and apparent manifestations, healthcare professionals can improve patient outcomes by promptly identifying and addressing medical conditions, regardless of their size or symptomatic presentation.

## Conclusions

The etiology and clinical sequelae of left atrial masses are variable but typically associated with potentially serious and life-threatening adverse events due to embolization. Initiation of surgical intervention is recommended to avoid unpredictable adverse events and improve the patient’s prognosis. Clinical suspicion serves as a crucial guidepost in medicine, prompting healthcare providers to consider potential diagnoses and pursue further investigation, even when initial findings may be ambiguous or nonspecific.

Therefore, in the pursuit of providing optimal care, it is imperative to embrace the possibility of rare occurrences, even when they may appear statistically unlikely. By cultivating a mindset of openness, curiosity, and clinical suspicion, healthcare professionals can uphold their commitment to delivering comprehensive and personalized care, ensuring that no significant condition goes unnoticed or undiagnosed.
